# OX40 agonist stimulation increases and sustains humoral and cell-mediated responses to SARS-CoV-2 protein and saRNA vaccines

**DOI:** 10.3389/fimmu.2022.896310

**Published:** 2022-09-27

**Authors:** Rebekka Duhen, Michael Beymer, Shawn M. Jensen, Srinivas Abbina, Suraj Abraham, Nikita Jain, Anitha Thomas, Andrew J. Geall, Hong-Ming Hu, Bernard A. Fox, Andrew D. Weinberg

**Affiliations:** ^1^Earle A. Chiles Research Institute, Providence Cancer Institute, Portland, OR, United States; ^2^Precision NanoSystems Inc, Vancouver, BC, Canada

**Keywords:** ANTI-OX40, co-stimulation, sa-RNA vaccine, T cell activation, SARS-CoV-2 vaccine, protein vaccine

## Abstract

To prevent SARS-CoV-2 infections and generate long-lasting immunity, vaccines need to generate strong viral-specific B and T cell responses. Previous results from our lab and others have shown that immunizations in the presence of an OX40 agonist antibody lead to higher antibody titers and increased numbers of long-lived antigen-specific CD4 and CD8 T cells. Using a similar strategy, we explored the effect of OX40 co-stimulation in a prime and boost vaccination scheme using an adjuvanted SARS-CoV-2 spike protein vaccine in C57BL/6 mice. Our results show that OX40 engagement during vaccination significantly increases long-lived antibody responses to the spike protein. In addition, after immunization spike protein-specific proliferation was greatly increased for both CD4 and CD8 T cells, with enhanced, spike-specific secretion of IFN-γ and IL-2. Booster (3^rd^ injection) immunizations combined with an OX40 agonist (7 months post-prime) further increased vaccine-specific antibody and T cell responses. Initial experiments assessing a self-amplifying mRNA (saRNA) vaccine encoding the spike protein antigen show a robust antigen-specific CD8 T cell response. The saRNA spike-specific CD8 T cells express high levels of GrzmB, IFN-γ and TNF-α which was not observed with protein immunization and this response was further increased by the OX40 agonist. Similar to protein immunizations the OX40 agonist also increased vaccine-specific CD4 T cell responses. In summary, this study compares and contrasts the effects and benefits of both protein and saRNA vaccination and the extent to which an OX40 agonist enhances and sustains the immune response against the SARS-CoV-2 spike protein.

## Introduction

SARS-CoV-2 has been the leading cause of death in the years 2020 and 2021 in many parts of the world. The novel coronavirus-19 disease (COVID-19) has had a tremendous impact on society and daily life at present and will for years to come. To curb the spread of SARS-CoV-2, vaccines were proposed as a path to reduce viral spread, infections, and hospitalizations. Based on previous research, geared towards personalized cancer therapies, SARS-CoV-2 spike protein mRNA vaccines were quickly developed and then tested to combat COVID-19 (1–6). While other companies with differing approaches followed suit (7, 8), the ability to quickly produce a vast amount of a highly effective vaccine has made the mRNA approach the most promising and widely distributed vaccine in the world. The vaccines were initially shown to be up to 95% effective at protecting against viral infection with the original strain. Comparing the two mRNA vaccines, the Moderna vaccine (mRNA-1271) appeared to produce slightly higher antibody titers at the 6-months mark, compared to the Pfizer/BioNTech (BNT162b2) vaccine. This is not entirely surprising, given the higher dose of the Moderna vaccine (100 µg versus 30 µg, respectively). While the results of the mRNA approach seem quite promising, we still do not know the longevity of the immune responses induced by these vaccines. Data showed that B cell immunity induced by both mRNA vaccines waned over time (9) and models implied that this decrease is observed with most SARS-CoV-2 vaccines (10). Based on these findings, booster immunizations, 5 months following the primary dose, have now been approved in the USA for all adults as well as children from 5 years of age (11), and immunizations of 6 month-4 year old children were added recently, using either Pfizer or the Moderna vaccine. While mRNA vaccines have been reported to be safe, adenoviral vector vaccines were shown to cause thrombosis in a small minority of recipients (12). Furthermore, new SARS-CoV-2 variants (such as Delta and Omicron and its subvariants) are posing a constant challenge to the current vaccines; however, mRNA vaccines can quickly be adapted and modified to encode new mutated sequences.

To bind and enter cells SARS-CoV-2 uses a homotrimeric spike protein to interact with a cell surface receptor, the angiotensin converting enzyme 2 (ACE2), which triggers a cascade of events allowing membrane fusion and viral entry into cells (13). The spike protein domain crucial for this interaction is the receptor binding domain (RBD), which is present within the S1 subunit (14). B cell responses, *via* the production of neutralizing antibodies, are important to prevent the binding of SARS-CoV-2 to cells within the body. In support of this, Cavazzoni et al. showed that follicular helper T cells (Tfh) are essential for optimization of the germinal center response to SARS-CoV-2 protein vaccines and affinity maturation (15).

In addition to the humoral response, a potent antigen-specific T cell response is also important to induce long-lasting anti-viral immune memory (16). CD4 and CD8 T cells typically encounter foreign antigen presented by activated antigen presenting cells (APCs), expand, contract, and then persist as memory cells. These memory T cells can quickly respond if the host is re-infected with the pathogen. While several co-stimulatory molecules are involved and necessary to generate a strong memory T cell response, our lab and others have demonstrated that OX40 agonists not only enhance effector T cell activation, but also increase the number of memory T cells that persist long-term (17, 18). OX40, a member of the TNF receptor family (TNFRF), is upregulated on CD4 T cells (and to a lesser extent on CD8 T cells) after antigen recognition. Once expressed on the surface of T cells, engagement of OX40 with its ligand, OX40L, on activated APCs results in increased T cell expansion, effector function and survival (19). Engagement of OX40 *in vivo* by agonist antibodies or fusion proteins can lead to higher levels of T cell cytokines, aid in viral clearance, and augment anti-tumor T cell responses (20). Furthermore, OX40 stimulated T cells cooperate with ICOS expressed on the Tfh cells, to increase the humoral immune response. This leads to production of higher affinity antibodies and long-lived memory B cells, which help to sustain antibody production in future pathogen encounters (21). Hence, engaging OX40 during an ongoing immune response can increase both CD4 and CD8 T cells as well as antibody responses.

In this study, we examined whether an OX40 agonist could enhance immune responses to both protein and saRNA COVID-19 vaccine platforms. We hypothesized that this strategy would lead to stronger T cell responses as well as increased antibody titers that may help to neutralize spike protein binding to the ACE2 receptor. T cell responses and the persistence of spike-specific antibodies were examined after prime-boost vaccination. We found that injecting an OX40 agonist in both the prime and boost was the most effective way to maintain higher antibody titers over a long period of time and this approach also amplified vaccine-specific CD4 T cell responses. When compared to protein immunization the saRNA vaccine induced a much stronger spike-specific CD8 T cell response, which was enhanced by OX40 agonist administration. Similar to protein vaccination, the OX40 agonist also increased a vaccine-specific CD4 T cell response in saRNA vaccinated mice. Thus, our findings show that an OX40 agonist can enhance both, protein and saRNA COVID-19 vaccine approaches.

## Results

### An OX40 agonist enhances SARS-CoV-2 spike protein vaccination

**Figure 1:**
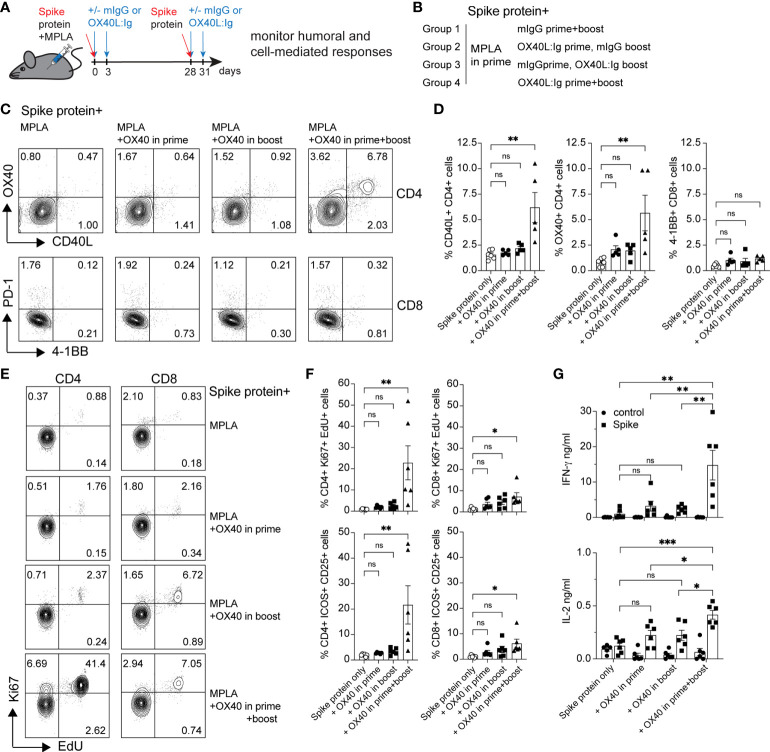
OX40 agonist enhances CD4 and CD8 antigen-specific T cell immunity to spike protein vaccination. Groups of mice were immunized with the SARS-CoV-2 spike protein emulsified in Montanide ISA 51 and MPLA (present in the prime), with mouse IgG (mIgG) or with OX40L:Ig injection in the prime, in the boost or both. Cells were isolated from the blood at 10 weeks post-immunization and cultured in the presence of recombinant spike protein. **(A)** Schematic representation of immunization with the spike protein and timing of the OX40L:Ig injections. **(B)** Description of the experimental group layout. **(C)** Expression of OX40 and CD40L on CD4 T cells (top) and PD-1 and 4-1BB on CD8 T cells (bottom) by flow cytometry after overnight incubation (16 hrs) with the spike protein. The dot plots shown are from representative mice in each group. Numbers in each quadrant indicate percent positive cells. **(D)** Summary of CD40L- and OX40-expressing CD4 T cells and 4-1BB-expressing CD8 T cells in all experimental groups. **(E)** Cells were cultured for 3 days in presence of antigen and EdU was added for 18 hrs to reveal replicating cells. Numbers in each quadrant indicate percent positive cells. **(F)** Ki67 expression and EdU incorporation (top panels) and ICOS/CD25 expression (bottom panels) were analyzed to assess T cell proliferation and activation in CD4 and CD8 T cells in all groups. **(G)** Supernatants of the 3-day cultures (blood) were assessed for the presence of IFN-γ and IL-2 in absence or presence of spike protein. N=6 animals per group, 1 of 2 experiments is shown. Each individual symbol in the bar graphs represents a single mouse. In **D**, **F** and **G**, bars indicate mean ± SEM. One-way ANOVA with Tukey’s multiple-comparisons test. *P<0.05, **P<0.01, ***P<0.001, ns, not significant.

Our laboratory has examined the effects of OX40 agonists enhancing T cell responses in both viral and cancer immunity for many years (22–25). We have generated a hexameric OX40L:Ig fusion protein (26), that has potent immune stimulatory effects in mice, non-human primates and humans (27). To examine the effect of OX40L:Ig to enhance protein vaccinations to SARS-CoV-2, we immunized mice with a prefusion stabilized trimeric spike protein (28). Mice were immunized with the spike protein and a TLR-4 agonist, monophosphoryl lipid A (MPLA), emulsified in Montanide ISA 51. In the boost, mice received the protein in PBS, without MPLA. Mice were treated with mouse IgG (mIgG) or OX40L:Ig in the prime vaccination, the boost vaccination or during both the prime and boost. The groups were boosted 28 days after the prime (**Figures 1A, B**). We first assessed the ability of the vaccine to activate T cells in the blood *via* ICOS/Ki67 upregulation on CD4 T cells, and GrzmB/Ki67 upregulation on CD8 T cells. CD4 and Treg cells were highly activated in the blood 5 days after the boost, with an average of 40.6% (± 15.9) Ki67+ICOShi CD4 T cells. CD8 T cells increased proliferation to a lesser extent and did not display a significant amount of GrzmB+Ki67+ cells (3.4% ± 2.6) (**Supplementary Figures 1A, B**). The activation of CD4 T cells was partially due to OX40 agonist alone in the absence of vaccine, as depicted in the OX40 agonist control group (14.0% ± 3.5). Thus, while upregulation of these activation markers on T cells could identify spike-specific T cells, we developed assays to enumerate the percentages of spike-specific T cells more accurately in the blood. Peripheral blood lymphocytes were isolated 6 weeks post-boost, and the cells were cultured with spike protein *in vitro*. After overnight incubation with the spike protein, CD40L and OX40 expression were analyzed on the surface of CD4 T cells, and PD-1 and 4-1BB on CD8 T cells (19, 29–31). CD40L and OX40 expression were detected on the surface of CD4 T cells from mice that had been injected with the OX40L:Ig fusion protein in both prime and boost, whereas the percentage of antigen-specific cells was significantly lower in animals receiving one injection of the OX40 agonist (in the prime or boost) and low in mice receiving vaccine alone (no OX40L:Ig) (**Figure 1C** top and **D**, left panel). In contrast to CD4 T cells we did not detect an increase in the percentage of 4-1BB+ CD8 T cells after ex vivo culture with the spike protein (**Figure 1C** bottom and **D**, right panel). In this short-term assay stimulation of CD4 T cells may be more efficient, as opposed to cross-presentation of peptides to CD8 T cells *via* MHC class I, which could take longer than 12-16 hrs to induce activation. Thus, we also analyzed the ability of the spike protein to induce T cell proliferation and cytokine secretion in 3-day cultures. While spike-specific CD4 T cells showed robust proliferation especially in mice receiving OX40L:Ig in the prime and boost, the CD8 T cells did proliferate, but not as vigorously (**Figures 1E, F**). When cytokines were assessed, the highest level of IFN-γ was observed in the supernatants of T cells isolated from mice treated with the OX40L:Ig protein in both the prime and the boost. IL-2 showed a similar pattern, albeit at much lower levels (**Figure 1G**). The Th2 specific cytokine, IL-4, was not detected in these cultures potentially due to the strong TLR agonist, MPLA, which tends to skew T cells towards a Th1 lineage phenotype (**Supplementary Figure 1C**).

### TLR4 and OX40 stimulation and their effects in prime versus boost

**Figure 2:**
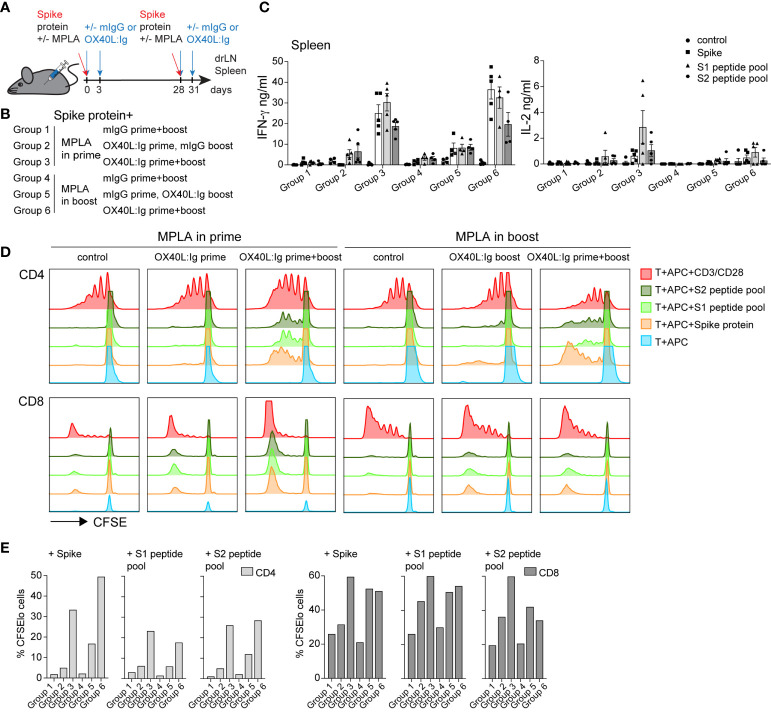
OX40 agonist enhances vaccine-specific T cell responses independent of whether a TLR4 agonist was administered in the prime or the boost. Groups of mice were immunized with the SARS-CoV-2 spike protein emulsified in Montanide ISA 51 with MPLA injected in the prime or boost. In groups 1-3, mIgG or OX40L:Ig were administered in the prime with MPLA or OX40L:Ig was given in both prime and boost. Groups 4-6 received MPLA in the boost with mIgG or OX40L:Ig or OX40L:Ig was given in both prime and boost. **(A)** Schematic representation of immunization with the spike protein, MPLA and timing of the OX40L:Ig injections. **(B)** Description of the experimental group layout. **(C)** Secretion of IFN-γ and IL-2 cytokines examined in the supernatant of splenocytes cultured for 3 days alone, in presence of spike protein, S1 or S2 spike peptide pools. Each data point represents an individual animal. Bars indicate mean ± SEM. **(D)** CFSE-labeled splenic T-cells, were cultured with irradiated APCs alone, pulsed with spike protein, or S1 and S2 spike peptide pools. CD3/CD28 stimulation was used as positive control for these experiments. Histograms show the cell division by dilution of CFSE as analyzed by flow cytometry after 4 days of culture, cells were gated on either CD4 or CD8 T cells. **(E)** Bar graphs showing the percentage of CFSE^lo^ CD4 and CD8 T cells in 4-day cultures after spike antigen stimulation. 1 of 2 experiments is shown in **(B–D)**.

We next addressed whether MPLA was more important for enhancing immune responses in combination with OX40L:Ig when delivered in the prime versus boost (see **Figure 2A, B**). Previous work from our group has shown that LPS, *via* TLR4 activation, can synergize with OX40 to enhance vaccines (17). MPLA, which also activates APCs *via* TLR4 uses TRIF signaling (instead of the MyD88 pathway) to release proinflammatory cytokines, is less toxic and skews T cells towards a Th1 phenotype (32, 33). Analyses of T cell activation in blood 5 days post-boost, showed that CD4 T cells were proliferating (Ki67+ICOS^hi^) when MPLA was injected in the prime with the OX40 agonist delivered in the prime and boost (17.1% ± 12.3) (**Supplementary Figures 2A, B****)**. However, the OX40 agonist had a more substantial effect on proliferation/activation when MPLA was injected during the boost (35.8% ± 15.7 Ki67+ICOS^hi^ cells). In contrast, CD8 T cells were only minimally activated when examined for Ki67 and GrzmB expression (2.9% ± 2.2 and 5.9% ± 8.8) (**Supplementary Figures 2A, B****)**. Proliferation was generally greatest when MPLA was administered during the boost, for Tconv, Treg, CD8 T cells, as well as B cells (**Supplementary Figure 2B**). In parallel, we assessed the presence of Tfh cells in the animals, based on their role in fostering potent antibody responses. We tracked their frequencies at baseline, pre- and post-boost and found that, in line with recent studies (15, 34) CXCR5+ PD-1 positive Tfh cells are increased post vaccination, most prominently when OX40L:Ig was administered in both, prime and boost **(** **Supplementary Figure 2C****)**.

Mice were sacrificed 21 weeks post-boost and spleen and draining lymph nodes (drLN) were analyzed. We found increases in Tfh cells in both the drLN and spleen when OX40L:Ig was administered in prime and boost **(** **Supplementary Figure 2D****)**. To further analyze the spike-specific CD4 but also CD8 T cells responses we cultured the spleen and drLN cells not only with the spike protein but also overlapping 15-mers that cover the full-length protein with 2 peptide pools (S1 and S2). Peptides can bind better to MHCI (and MHCII) molecules and hence can be presented more efficiently to CD8 T cells. Supernatants from the splenocyte cultures incubated with these antigens were assessed for IFN-γ, IL-2 and IL-4 cytokine secretion. Antigen-specific IFN-γ production was significantly higher when the OX40 agonist was injected in both the prime and boost independent of whether MPLA was delivered in the prime or the boost (**Figure 2C**). Levels of IL-2 were also increased in the same groups, but IL-4 was very low to undetectable in all groups (**Figure 2C** and **Supplementary Figure 2E**).

We next assessed spike-specific T cell proliferation *via* CFSE dilution assay. This assay allowed us to determine proliferation in the absence of endogenous APCs that might harbor spike-specific peptides. T cells were purified and pooled from each treatment group and cultured with irradiated splenocytes isolated from naïve mice, pulsed with the spike protein or peptides. CFSE dilution of CD4 T cells 4 days after antigen stimulation revealed that proliferation was amplified in mice injected with the OX40L:Ig protein. CD4 T cell proliferation was greatest when mice received the OX40 agonist in both the prime and boost. Mice receiving vaccine with two doses of OX40L:Ig displayed an 18-30-fold increase compared to vaccine/MPLA alone (**Figures 2D, E****)**. CD8 T cells proliferated more vigorously in the presence of peptide pools and proliferated the strongest when MPLA was administered in the prime with two doses of the OX40 agonist. The fold-increase of CFSE dilution in CD8 T cells (OX40L:Ig in prime and boost versus control mice) was lower (2 to 3-fold) than in CD4 T cells (**Figure 2E**).

In summary, MPLA increased T cell activation and proliferation in the blood when delivered after the boost and this effect was accentuated when combined with the OX40L:Ig protein. However, vaccine-specific CD4 and CD8 T cell responses were elevated when the OX40 agonist was injected in both the prime and boost, independent of whether MPLA was administered in the prime or the boost.

### OX40 stimulation increases the longevity of vaccine-specific antibody responses

**Figure 3:**
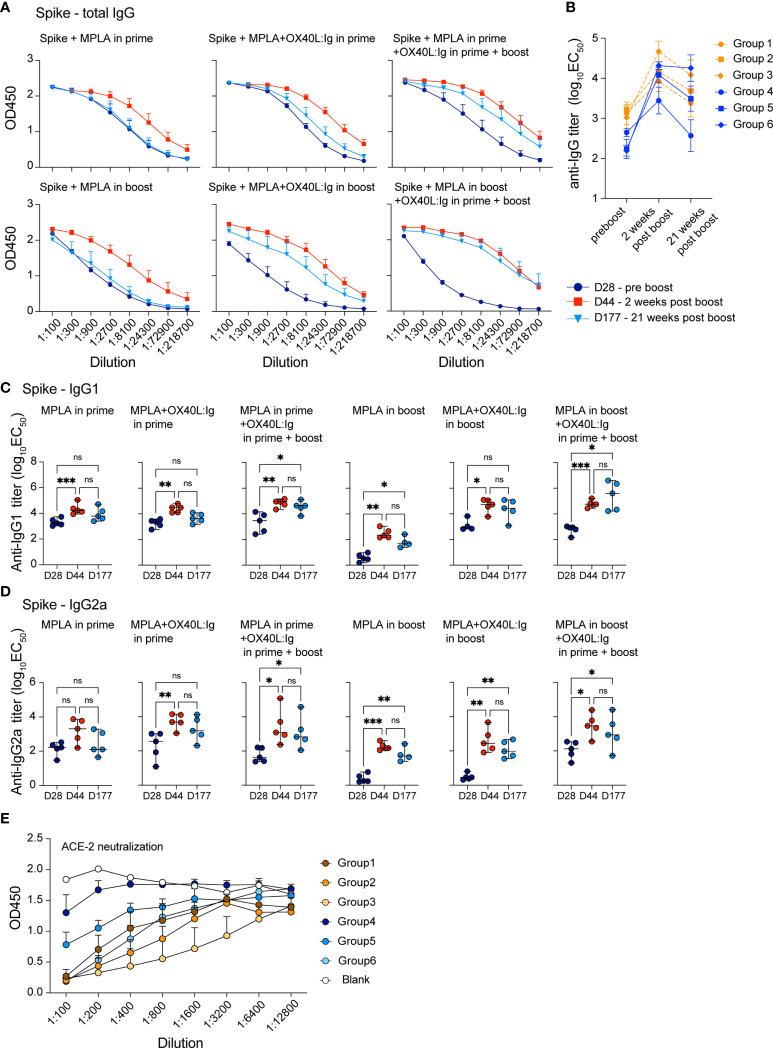
OX40 agonists increase the magnitude and longevity of spike vaccine-specific antibodies. Experimental scheme and groups are outlined in **Figures 2A, B**. Mice were bled on day 28, prior to the boost (indigo circles); day 44, 2 weeks post-boost (red squares); day 177, 21 weeks post-boost (blue triangles), and antibody titers to the spike protein were assessed. **(A)** shows total IgG titers against the spike protein. Values on the y-axis indicate absorbance measured at 450 nm. D=day **(B)** shows the log_10_(EC_50_) titers of all groups at each timepoint pre boost, two weeks post boost and 21 weeks post boost **(C)** shows the log_10_(EC_50_) titers of IgG1 isotype spike-specific antibodies and **(D)** shows the log_10_(EC_50_) titers of IgG2a isotype spike-specific antibodies. C and D, one-way ANOVA with Tukey’s multiple-comparisons test. *P<0.05, **P<0.01, ***P<0.001, ns, not significant. **(E)** Data from the neutralization assay performed using the ACE2-Fc protein and supernatants from all groups 7 weeks post boost. All samples were diluted 1:100 and serially diluted 1:3 or 1:4 eight times in the assay and the serial dilutions are indicated by connecting lines.

Vaccine-specific memory T and B cell responses are important to ensure long-term protection against the viral challenge. CD4 T cells help elicit and mature a potent B cell response which results in antibody production, affinity maturation and formation of long-lived plasma cells. The OX40L:Ig fusion protein greatly increased the vaccine-specific CD4 T cell response and induced Tfh cells (**Figures 1**, **2**), hence we hypothesized that vaccine-specific antibodies would also be increased by OX40 stimulation. We tested this hypothesis by assessing antibodies after serial blood draws in the experiments described above, to monitor the magnitude and kinetics of vaccine-specific antibodies. **Supplementary Figures 3A, B** illustrate the presence of spike- and RBD-specific antibody titers in the serum, which persisted up to 15 weeks post-boost. Spike- and RBD-specific antibody titers were observed in all mice immunized with the spike protein and when the OX40 agonist was injected in both the prime and boost, antibody levels were higher and more sustained (**Supplementary Figures 3A, B**, bottom; **C**, right). Quantification of antigen-specific antibodies using log_10_EC50 values shows the strongest antibody responses were found 2 weeks post-boost and the titers remained elevated when the OX40 agonist was injected in both the prime and boost (**Supplementary Figure 3C**). We next explored antibody titers in spike protein vaccinated mice receiving MPLA in the prime versus boost w/wo an OX40 agonist (pre-boost, 2 weeks, and 21 weeks post-boost). In mice immunized with spike protein + MPLA (no OX40 agonist) total IgG levels were relatively low 2 weeks post-boost and returned to pre-boost levels by 25 weeks. Spike protein immunization in combination with MPLA + OX40 agonist in the prime or MPLA + OX40 agonist in the boost showed longer-lived antibody responses (**Figure 3A**). Interestingly, when MPLA was delivered in the prime or boost, with two injections of the OX40 agonist, the spike-specific antibody levels did not return to prime levels and remained elevated for 25 weeks (**Figure 3A**, right). **Figure 3B** displays the change in titers over time.

Since MPLA is known to skew the immune response towards a Th1 phenotype, we further dissected the antibody isotype composition in these treatment groups. We assessed whether IgG1 (mainly Th2 response) or IgG2a (mainly Th1 response) isotypes predominated in any of these conditions. Analyses of all 6 groups showed that spike-specific IgG1 antibody levels were similar under most conditions, with MPLA/OX40 agonist in prime/boost groups displaying highest long-lived antibody titers post-boost (**Figure 3C**). In accord with a previous report (35), MPLA as an adjuvant in the prime increased IgG2a spike-specific antibodies, whereas levels remained lower when MPLA was injected in the boost, w/wo OX40 agonist administration (**Figure 3D**). IgG2a titers were comparable when the OX40 agonist was administered in the prime and boost, independent of whether MPLA was injected in the prime or boost and the OX40 agonist increased the longevity of the IgG2a response (**Figure 3D**). To address whether the serum antibodies had the ability to interfere with the interaction between ACE2 and the spike protein, we assessed their neutralizing activity. In line with our results above, the most potent activity was observed when OX40L:Ig was given in prime and boost – with MPLA in the prime **(** **Figure 3E****)**.

In conclusion, we found that OX40 agonist injections strongly augmented the magnitude and persistence of the vaccine-specific antibodies in mice vaccinated with the spike protein.

### OX40L:Ig injection when combined with a booster immunization amplifies vaccine-specific T cell responses

**Figure 4:**
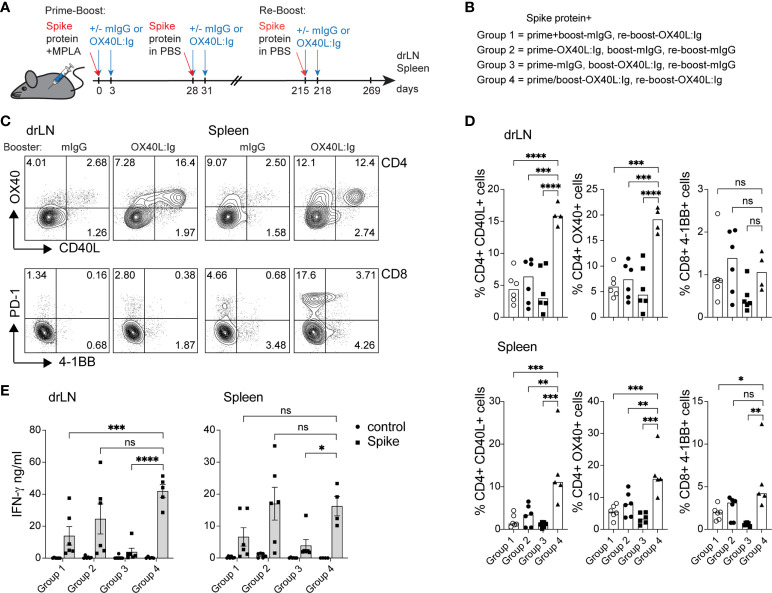
Booster immunization in combination with an OX40 agonist can increase T cell activation, antigen-specific CD4 T cells, and cytokine production. Mice initially immunized with the spike protein+MPLA in the prime received the OX40L:Ig in prime, boost or both. Animals were then boosted 30 weeks later. **(A)** Schematic representation of the booster immunization with spike protein w/wo OX40L:Ig injections. **(B)** Description of the experimental group layout. **(C, D)** Cells from drLN and spleen were isolated 7 weeks after the booster and examined for antigen-specific CD4 and CD8 T cells. **(C)** Flow cytometric analysis of the expression of CD40L and OX40 on CD4 T cells (top) and PD-1 and 4-1BB on CD8 T cells (bottom) after overnight incubation with the spike-protein (one representative mouse from each group is shown). Numbers in each quadrant indicate percent positive cells. **(D)** Bar graphs showing the summary of the percentages of CD40L+ CD4, OX40+ CD4 and 4-1BB+ CD8 T cells in the drLN and spleens of mice receiving booster immunizations ± OX40L:Ig. **(E)** Cytokine assessment for IFN-γ and IL-2 in the supernatant of 3-day cultures from drLN and spleen. N=5-6 animals per group, 1 of 2 experiments is shown. Each individual symbol in the bar graphs represents a single mouse. Bars indicate mean ± SEM. One-way ANOVA with Tukey’s multiple-comparisons test. *P<0.05, **P<0.01, ***P<0.001, ****P<0.0001, ns, not significant.

While spike-specific memory T cell responses were detected in most mice after prime-boost vaccination, we wanted to examine the effect of a booster vaccination in mice 7 months post-initial immunization. In the booster experiment, all mice were injected with the spike protein. The OX40 agonist was given to mice that had not previously received any OX40 stimulation and to mice that were previously injected with OX40L:Ig in both the prime and boost. The groups that received OX40L:Ig in either the prime or boost initially, received only mouse IgG in the re-boost. **Figures 4A, B** illustrate the layout of this experiment. **Supplementary Figure 4A** depicts early activation of Tconv, Treg and CD8 T cells 5 days post-booster immunization, and while OX40L:Ig fusion protein did increase activation, the vaccine alone also increased T cell activation. Interestingly, CD8 T cells proliferated more vigorously than after the initial prime/boost (**Supplementary Figure 4B**). We then assessed the antigen-specific T cell response in the spleen and drLNs of mice receiving the booster immunization. Mice were sacrificed 7 weeks after the booster and analyzed for spike protein-specific CD4 and CD8 T cells. In the short-term antigen-specific assays, both CD40L and OX40 were upregulated on CD4 T cells, in the drLN and spleen. Mice that received the OX40 agonist in both the prime/boost as well as in the booster immunization had the greatest vaccine-specific T cell response (**Figures 4C, D****)**. T cells isolated from animals receiving anti-OX40 in the boost and mIgG in the booster responded but to a lesser extent than the other groups (**Figures 4C, D****)**. The magnitude of the antigen-specific CD8 response was lower overall compared to CD4 T cells, but higher numbers of antigen-specific CD8 T cells were found in the spleen than in the drLN and OX40L:Ig accentuated this increase, as revealed by 4-1BB upregulation (**Figure 4D**, bottom right). T cell activation and proliferation were then assessed in cells cultured for three days in the presence or absence of the spike protein. CD4 T cells proliferated strongly in both drLN and spleen cultures, while CD8 T cell responses were lower in both tissues (**Supplementary Figure 4C**). We also examined cytokines that were produced by these T cells and found increased spike-specific IFN-γ in most conditions, compared to no antigen controls. Group C (no OX40L:Ig in the booster) had the lowest cytokine production, which was a consistent finding when compared to the other assays (**Figure 4D**). The greatest cytokine production was found in T cells isolated from mice receiving OX40 stimulation with the booster (groups B&D) (**Figure 4E**).

Thus, boosting mice increased the vaccine-specific T cell responses especially in mice that were injected with an OX40 agonist in the initial prime/boost and adding OX40 stimulation in the booster appears to further increase the vaccine-specific CD4 and CD8 T cell responses.

### OX40 agonist stimulation increases spike-specific T cell responses to a self-amplifying mRNA vaccine

**Figure 5:**
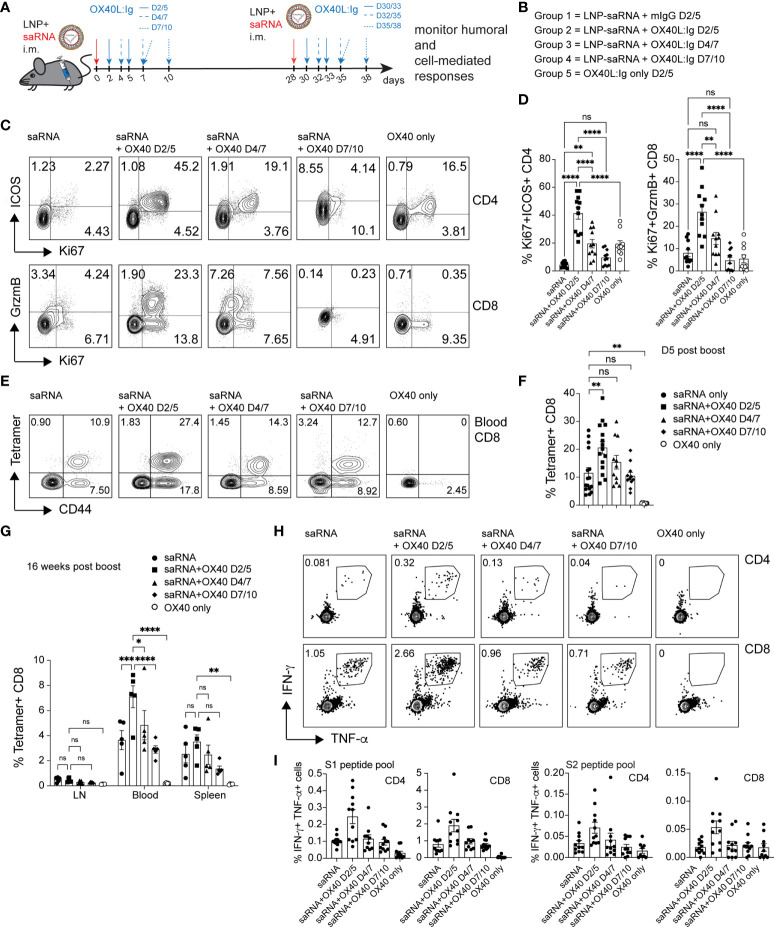
OX40 agonists enhance T cell responses in mice immunized with a self-amplifying RNA vaccine. Groups of mice were immunized i.m. with self-amplifying RNA (saRNA), encoding the spike-protein, encapsulated in lipid nanoparticles (LNPs). Mice received the vaccine alone or with OX40 stimulation on days 2/5, days 4/7 or days 7/10 post-vaccination. **(A)** Schematic representation of the timing of the administration of the saRNA vaccine w/wo OX40L:Ig. **(B)** Description of the experimental group layout. D=day **(C)** Flow cytometric assessment of the upregulation of ICOS/Ki67 on peripheral blood CD4 T cells and GrzmB/Ki67 on CD8 T cells five days after the 1^st^ OX40L:Ig dose. The dot plots shown are from representative mice in each group. Numbers in each quadrant indicate percent positive cells. **(D)** Summary of the percentage activated Ki67+ICOS+ Tconv CD4 cells and Ki67+GrzmB+ CD8 T cells in blood. Bars indicate mean ± SEM. **(E)** Flow cytometric assessment of the frequency of tetramer+ CD44+ CD8 T cells 5 days post-boost (in each group). Each plot is from a representative animal per group. Numbers in the upper two quadrants represent the tetramer+ cells. **(F)** Summary of the data in **(E)**. **(G)** Summary of tetramer+ T cells in blood, LN and spleen, 16 weeks post-immunization (day118). **(H)** Mice were euthanized at day124. Frequency of IFN-γ+ and TNF-α+ CD4 and CD8 T cells among splenocytes after *in vitro* stimulation with the S1 peptide pool. One representative animal per group is shown. The outlined box indicates the IFN-γ+TNF-α+ double-positive cells. **(I)** Summary of the frequency of IFN-γ+TNF-α+ CD4 and CD8 T cells stimulated with either S1 or S2 peptide pools *in vitro*. Bar graphs show the mean frequencies ± SEM per group in D, F, G and **(I)** One-way ANOVA with Tukey’s multiple-comparisons test. *P<0.05, **P<0.01, ***P<0.001, ****P<0.0001, ns, not significant. N=10-11 animals per group in **D, F, G**, and **(I)** 1 of 2 experiments is shown.

RNA vaccines have been approved for use in humans and have elicited both, vaccine-specific antibodies, and CD4 and CD8 T cell responses (16). Furthermore, in contrast to protein vaccines, mRNA vaccines have the potential for rapid, scalable manufacturing, owing to the high yields of *in vitro* transcription reactions (36). Hence, we wanted to test whether an OX40 agonist could also enhance immune responses to RNA vaccines. To test this hypothesis, we immunized mice with a self-amplifying mRNA, encoding an alphaviral replicase (to enable replication upon uptake in the cell cytoplasm) and the SARS-CoV-2 spike protein encapsulated in lipid nanoparticles (LNPs). SaRNA can provide high expression levels and simultaneously induce a strong innate immune response. Mice received two injections of 1 µg of saRNA (in prime and boost), 28 days apart, with or without the OX40L:Ig fusion protein. Proteins encoded by saRNA vaccines can take a few days to be produced and expression can be maintained for up to a month (37). Hence, the initial experiment assessed the optimal timing of OX40 agonist administration after saRNA vaccination (days 2/5, 4/7 and 7/10) (**Figures 5A, B**). Stimulation with the OX40L:Ig agonist, in absence of the saRNA vaccine, served as a negative control for these experiments. The activation status of T cells stimulated with the saRNA w/wo OX40 agonist was initially assessed 5 days after the boost. Mice that received OX40 stimulation at days 2/5 had the greatest level of CD4 T cell activation (41.27% ± 4.1, ICOS+Ki67+) and expression of these two makers declined in mice receiving the OX40 agonist at later timepoints (**Figures 5C, D****)**. The CD8 T cells expressed very high levels of GrzmB and Ki67 (26.42 ± 3.2, GrzmB+Ki67+) when the vaccine was combined with OX40 stimulation especially at days 2/5 after immunization (**Figures 5C, D****)**. Interestingly, this GrzmB+Ki67+ CD8+ cell phenotype was not observed when mice were immunized with protein (**Supplementary Figures 1A, 2A**). Since the activation data suggested that spike-specific CD8 T cell responses may be increased with the saRNA approach, we assessed the presence of spike-specific CD8 T cells after saRNA vaccination. A p:MHCI tetramer was used to quantify the vaccine-specific CD8 T cell response (an immunodominant epitope of the spike protein in C57BL/6 mice) (38). Tetramer+ CD8 T cells were enumerated in peripheral blood 5 days after the boost OX40L:Ig injection. 11.5% ( ± 1.9) of peripheral blood CD8 T cells were tetramer+ in mice injected with saRNA vaccine alone. Mice injected with the OX40 agonist on days 2/5 showed an increase in tetramer^+^ CD8 T cells (20.58% ± 2.2) compared to mice receiving vaccine alone. When the OX40 agonist was delivered at later time points the enhancement of vaccine-specific responses was lower (15.4% ± 2.4 and 10.44 ± 1.3 tetramer+ cells, respectively) (**Figures 5E, F****)**. The same analysis was repeated 16 weeks post-vaccination, when the mice were sacrificed, to monitor contraction of the CD8 T cell response in the LNs, spleen, and blood. Total memory CD8 T cells were lower in the lymph node (1.3%) and ranged on average between 9-13% in blood and spleen, with no significant differences between the groups (**Figure 5G**). As for tetramer^+^ CD8 T cells (16 weeks post-vaccine), a similar trend to day 5 post-boost was observed. Mice injected with OX40 agonist at days 2/5 still contained the highest percentage of tetramer^+^ CD8 T cells (2-fold above vaccine alone). However, compared to day 5 post-boost in the blood, the cells had contracted approximately 4-fold in each group (**Figure 5G** and **Supplementary Figure 5A**). Interestingly, when the frequency of spike-specific tetramer^+^ CD8 T cells in mice immunized with the protein vaccine were evaluated in peripheral blood at the same time point (16 weeks post-boost) little to no tetramer^+^ CD8 T cells were detected (**Supplementary Figure 5B**). Splenocytes followed a similar trend, but LNs contained much lower percentages of tetramer^+^ CD8 T cells (**Figure 5G** and **Supplementary Figure 5A**). Production of IFN-γ and TNF-α was also assessed upon stimulation with spike peptide pools in the saRNA vaccinated mice. Both CD4 and CD8 T cells produced IFN-γ and TNF-α and the highest percentage of IFN-γ^+^TNF-α^+^ positive cells were found in the day 2/5 OX40 agonist treated group (**Figure 5H**). In general, the frequency of IFN-γ secreting cells was higher in CD8 T cells when compared to CD4 T cells (**Figure 5I**). Proliferation of CD4 and CD8 vaccine-specific T cells was also assessed by CFSE dilution. Mice injected with saRNA alone increased spike protein-induced CFSE dilution in CD4 and CD8 T cells compared to the negative control group (OX40 stimulation alone). When the OX40 agonist was delivered on days 2/5 and 4/7 there was an increase in the percentage of T cells that diluted CFSE when compared to mice receiving vaccine alone (**Supplementary Figures 5C, D****)**. Similar to the results from the protein vaccine experiment (CFSE dilution; **Figures 2D, E****)**, the OX40L:Ig induced the greatest fold-increase in spike-specific CD4 proliferation. It also increased CFSE dilution in CD8 T cells from mice vaccinated with saRNA, but to a lesser extent than in the CD4 cells. Interestingly, the magnitude of the CD4 response (maximum percentage of diluted cells) was a slightly higher in the protein versus saRNA vaccinated mice. Finally, the serum antibody levels in saRNA immunized groups were examined, prior to the boost and two- and 12-weeks post-boost. We found that, in contrast to the protein vaccine, spike-specific antibodies generated with the saRNA only and saRNA + OX40 stimulation at days 2/5 or 4/7 were predominantly of the IgG2a isotype, with lower IgG1 titers (**Supplementary Figure 5E**). OX40 agonist delivered late after vaccination at days 7/10 had almost no effect on either isotype and titers were low overall.

Taken together, the data suggests that the saRNA vaccine approach generates a strong Th1-type response with long lasting, potent spike-specific CD8 T cells and this response can be enhanced by OX40L:Ig administration shortly after immunization.

## Discussion

The SARS-CoV-2 pandemic has been ongoing for two and a half years and in several countries individuals have received two vaccine doses, followed by one or two boosters. In order to reduce or eliminate the need for recurrent immunizations we tested whether an OX40 agonist would increase the immune response to a SARS-CoV-2 vaccine. This idea has been supported by previous publications showing that OX40 agonists can have strong immune adjuvant effects in the generating vaccine-specific T cells in mice, monkeys, and humans (22, 25, 39). OX40 agonists have been injected systemically to hundreds of cancer patients with low toxicity (22, 23), hence this is a strategy that could potentially be translated to human vaccines. Several studies suggest that co-stimulation *via* OX40 in a vaccine setting can not only increase CD4 T cell activation but can also boost CD8 T cell responses and increase secretion of vaccine-specific antibodies (24, 40, 41). The timing of OX40 agonist administration combined with vaccination has been explored, with findings showing that OX40 agonist injections delivered during the vaccine boost (14 days after a priming immunization) resulted in increased long-lived polyfunctional CD4 and CD8 T cells (39). However, another study showed that if the OX40 agonist was delivered after protein vaccination or viral challenge it could dampen immune responses (42).

In this study, we found that an OX40L:Ig fusion protein greatly increased vaccine-specific CD4 T cell responses and augmented spike-specific antibody secretion when injected with a protein vaccine. Interestingly, the saRNA vaccine was much more effective at generating spike-specific CD8 T cells, which was accentuated by the OX40L:Ig agonist.

Based on the differences observed in the publications discussed above, we examined the timing of OX40 agonist administration (OX40L:Ig in prime, boost or both) in combination with a spike protein vaccine. Panagioti et al. observed that if an OX40 agonist was injected during the booster phase of vaccination, it was more efficient to enhance T cell immunity than if it were administered during the priming phase (39). When we analyzed T cell activation 5 days after the boost, the OX40 agonist administered with the boost induced stronger proliferation in the CD4 T cell compartment than when given during the prime. However, contrary to the Panagioti manuscript, we detected the greatest increase of vaccine-specific T cell percentages when the OX40 agonist was injected in both the prime and the boost (**Figures 1** and **2B**). Differences between the two studies included the use of synthetic long peptides for vaccination by Panagioti et al, while we vaccinated with a whole protein; the time interval between prime and boost, which was 14 days, whereas in our study it was 28 days; and lastly we used the OX40L:Ig protein versus an OX40 agonist antibody (OX86) used in their study.

Successful SARS-CoV-2 vaccines should elicit long-lasting protection (via memory T and B cell responses), which will allow us to live with this virus in an endemic phase. In this study we explored how to increase the longevity of the vaccine-specific immune response by co-administration of an OX40 agonist with two different SARS-CoV-2 vaccines. We assessed the presence of spike-specific T cells and antibodies in mice several months after prime/boost immunizations w/wo the OX40L:Ig protein. Injecting the OX40 agonist in both the prime and the boost delivered the greatest numbers of functional spike-specific CD4 T cells as well as Tfh cells. Similarly, mice subjected to two rounds of OX40 agonist stimulation showed the greatest increase in spike- and RBD-specific antibody titers in their serum, and these levels were sustained for 6 months. This is an important observation because antibody responses elicited by the currently approved vaccines wane 3-6 months after the boost immunization (9). Thus, injecting the OX40 agonist in the prime and boost appears to increase the longevity of vaccine-specific immunity, which would potentially alleviate the need for repeated booster immunizations.

When vaccinated mice were re-challenged with a booster immunization 7 months later, they mounted an increased T cell response. OX40 engagement further increased the levels of activated and proliferating CD4 and CD8 T cells after the booster (**Supplementary Figure 4**). When analyzing the spike-specific immune responses in drLN and spleen several weeks after the booster it was clear that delivering the OX40L:Ig protein with all immunizations (prime/boost/booster) elicited the highest levels of spike-specific CD4 and CD8 T cells. These data suggest that an OX40 agonist added to a booster vaccine could enhance spike protein-specific immunity in individuals already immunized against the spike protein.

While mice injected with the spike protein vaccine combined with an OX40 agonist elicited a strong vaccine-specific antibody response (**Figure 3**), the CD8 T cell response was not very robust. Since RNA vaccines are known to elicit a potent CD8 T cell response, we determined whether an OX40 agonist could enhance CD8 responses when combined with a SARS-CoV-2 mRNA vaccine (16, 43, 44). mRNA vaccines have some advantages over protein vaccines, as they are easier and faster to produce as well as scalable for large immunization studies (36). In particular for this study we tested whether an OX40 agonist could enhance a self-amplifying RNA vaccine which contains an alphaviral replicase together with a gene of interest (spike protein) that is encapsulated in lipid nanoparticles (45). The saRNA vaccines require a much lower dose (0.1-1 µg/dose) compared to “traditional” mRNA vaccines (30-100 µg/dose), while at the same time leading to expression of higher protein levels (37). In our experiments, the saRNA-LNP vaccine was able to generate a strong vaccine-specific CD8 T cell response when compared to protein vaccination, and the antibody titers were biased towards a Th1-type response. Of importance for this study, the OX40 agonist was able to increase T cell activation and the magnitude of antigen-specific CD4 and CD8 T cell responses. In particular, the vaccine-specific CD8 T cell response elicited by the saRNA vaccine alone was strong (20% tetramer+ cells) at the peak of activation and the OX40-agonist increased it approximately 1.5 to 2-fold. The vaccine-specific CD4 T cell response to the saRNA vaccine was lower than with protein vaccination; however, the OX40-agonist did increase the CD4 T cell response to the saRNA vaccine as assessed by both proliferation and cytokine production (**Figure 5**).

It is clear from these studies that OX40 agonists can enhance vaccine-specific immune responses for both protein and RNA vaccines. In particular, both the magnitude and longevity of vaccine-specific responses were increased by the OX40L:Ig fusion protein especially when delivered both in the prime and the boost. A recent study combined an OX40 agonist antibody with a Sindbis alphavirus vector that expressed the spike protein and they found an enhancement of T cell activation, cytokine production and antibody titers (46). In most experiments however, mice were analyzed at only 7 days after the prime for metabolic and transcriptional changes, effects on T cell subsets, secretion of cytokines and cell surface markers. The authors assessed activation of total T cell populations with little distinction for vaccine-specific T cells, which differs from our study. Also, in this study we evaluated the longevity of the B and T cell responses, which was not emphasized in their study.

While both studies show that OX40 agonists can enhance COVID-19-specific vaccines, injecting a protein OX40 agonist is not practical for delivery for human vaccines. Engineering the OX40L:Ig fusion protein into viral vaccine vectors or adding the OX40L:Ig mRNA into the same lipid nanoparticles as RNA vaccines would be an economical and practical approach that will be pursued by our group in the future.

The data obtained with both protein and saRNA vaccines in these studies, suggest that a heterologous prime/boost approach may be beneficial to increase the lack of a CD8 response in the protein setting. Heterologous prime/boost vaccinations have been performed in humans for the spike protein (e.g. adenoviral vector vaccines + mRNA vaccines, or mRNA-Pfizer, followed by mRNA-Moderna) and this approach has officially been accepted by the government authorities. A heterologous and efficient prime-boost setting has been reported decades ago for HIV vaccines (47) and has since gained momentum for a wide range of pathogens. Heterologous vaccination can foster a broader array of immune responses (e.g. Th1 versus Th2), increase the effectiveness of existing vaccines and induce more immunogenic responses. It can lead to higher neutralizing antibody titers and more potent T cell responses compared to using the same vaccine in the prime-boost setting (48). While this suggests that heterologous prime/boost may lead to superior immune responses, careful analysis of combined approaches in the COVID-19 vaccine setting, is lacking. Our data suggest that there are differences in the potency of generating humoral and cell-mediated immune responses with the protein and RNA vaccine approaches. Of course, there are limitations - the assessment of the T cell and antibody responses in mice does not always correlate with level of protection in humans. Therefore, future experiments will combine both vaccine approaches, together with increased T cell costimulation. In summary, we observed differential immune stimulating effects when comparing protein versus saRNA SARS-Cov2 vaccines and it is clear that OX40 agonists can enhance immunity to both approaches.

## Material and methods

### Animals

C57BL/6 mice were purchased from the Jackson Laboratory. All animals were bred and maintained under specific pathogen-free conditions in the Providence Portland Medical Center (Portland, OR) animal facility and all experiments were performed in accordance with the guidelines of the Institutional Animal Care and Use Committee. Only females were used for the COVID vaccination studies. Control polyclonal mouse IgG was purchased from BioXCell. Anti-OX40L:Ig mAb was produced by MedImmune/AstraZeneca. Animals were randomly assigned to treatment cohorts. No outliers were excluded from the data presented.

### Synthesis of self-amplifying mRNA encoding the SARS-CoV-2 spike protein

The codon-optimized gene, encoding the full-length spike protein of SARS-CoV-2 with a single dominant mutation was synthesized and cloned into Precision NanoSystem’s proprietary custom self-amplifying mRNA cloning vector. The vector incorporates non-structural proteins encoding VEEV alphavirus replicases and a strong sub-genomic promoter with an engineered multiple cloning site. The cloned codon-optimized genes were synthetically constructed and amplified in Escherichia coli and purified using a Plasmid Maxi kit (QIAGEN). High quality saRNA was synthesized using a proprietary manufacturing process developed by PNI. Briefly, the plasmid DNA was linearized by restriction digest at the 3′ end of the saRNA sequence. Next, the linearized DNA templates were transcribed into RNA using the cell-free *in vitro* transcription and enzymatic capping method described by Geall A et al. (49).

### Formulation of lipid nanoparticles encapsulating saRNA

A lipid mix was prepared at a concentration of 37.5 mM in ethanol using a proprietary ionizable lipid, 1,2-distearoyl-sn-glycero-3-phosphocholine, cholesterol and 1,2-dimyristoyl-rac-glycero-3-methoxypolyethylene glycol-2000. saRNA was diluted to a concentration of 252 μg/ml in RNA formulation buffer at pH 4. Lipid mix in ethanol and saRNA in aqueous buffer were mixed to form lipid nanoparticles using a NanoAssemblr^®^ Ignite™ NxGen™ microfluidic mixer at a flow rate ratio of 3:1 (saRNA to lipids), total flow rate of 12 ml/min and start waste volumes of 0.35 ml and end waste volume of 0.05 ml. The LNP were then diluted 40× in PBS (Ca^2+^ and Mg^2+^ free) and further processed using Amicon^®^ ultra 15 10kDa MWCO units (EMD Millipore) filtration technique at 2000 x g for 30 min at 4°C to remove ethanol. The final LNPs were mixed with a PNI proprietary cryobuffer (1:1 V/V) and stored at -80°C. The LNPs were thawed at RT before immunization of animals.

### LNP characterization

After preparation of LNPs as described above, particle size (hydrodynamic diameter of the particles) was determined by Dynamic Light Scattering (DLS) using a ZetaSizer™ Nano ZS™ (Malvern Instruments, UK). He/Ne laser of 633 nm wavelength was used as the light source. Data were measured from the scattered intensity data conducted in backscattering detection mode (measurement angle = 173°). Measurements were an average of 10 runs of two cycles each per sample. Z -average size was reported as the particle size and is defined as the harmonic intensity averaged particle diameter. All solutions were analyzed using polystyrene cuvettes.

### saRNA encapsulation efficiency

saRNA encapsulation efficiency (EE%) was measured by a modified Ribogreen™ assay (Quanti-iT RiboGreen™ RNA assay kit, Fisher).1x TE buffer, and a 2% Triton-X (*w*/*v*) in 1x TE buffer were prepared for diluting the LNPs to the required concentrations for the assay. A 20 ug/mL RNA stock solution was prepared using 1x TE. Solutions for standard curve were prepared in the range of 0.1 – 2 ug/mL in Triton-X TE buffer in a 96 well. LNP solutions were then diluted in the 96 well plate using 50 ul 1x TE or 2% Triton-X TE buffer and incubated for 10 min at 37 °C (total 100 uL). 100 μL of 1:100 diluted Ribogreen reagent in 1x TE was then added to the wells and gently agitated for 30 sec. saRNA concentrations were quantified by measuring fluorescence (λem = 525 nm, λex = 485nm) at room temperature using a BioTek™ Synergy™ H1 Hybrid Multi-Mode Monochromator™ Fluorescence Microplate Reader. Encapsulation efficiency (EE) was calculated using the following equation:

EE= 100x {(Total RNA_(RNA in Triton TE)_ - RNA outside LNP_(Ribogreen in TE)_)/Total RNA_(Ribogreen in Triton TE)_}

### Immunization

Animals were immunized s.c. in the right flank/inguinal area of the animal with an emulsion of Montanide ISA 51 with 20 µg/ml of spike protein (Lake Pharma, Inc) with or without Monophosphoryl Lipid A (MPLA) (Avanti Polar Lipids, Inc) as adjuvant. Additionally, the animals received anti-OX40L:Ig injected i.p. on days 0 and 3 post immunization, or mIgG. Mice were boosted with 20 µg/ml of spike protein in PBS, with or without MPLA. saRNA-LNP preparation was obtained from PNI. Animals were immunized i.m. in the left or right footpad with 1 µg of saRNA in lipid nanoparticles, diluted in 50 µl of PBS. Animals also received two injections of anti-OX40L:Ig or mIgG, i.p., on differing days post immunization, 3 days apart.

### Blood collection and lymphocyte isolation

Blood was isolated from the animals by bleeding from the saphenous or submandibular veins. Between 0.1 and 0.4 ml of blood were collected, depending on the downstream applications. When mononuclear cells were isolated and analyzed, blood was collected in heparin-coated tubes. For T cell activation assays, peripheral blood mononuclear cells were separated using Fico/Lite-LM (R&D Systems) mouse cell separation medium. The peripheral mononuclear cells were washed with complete RPMI 1640 (Gibco) containing 0.292 ng/ml glutamine, 100 U/ml streptomycin/penicillin, 0.1 μM nonessential amino acids, 1 mM sodium pyruvate, and 10 mM HEPES (Sigma-Aldrich) and used in further experiments. For assessing cell phenotypes in blood, the blood was incubated with the ammonium-chloride-potassium (ACK) buffer to lyse red blood cells, prior to staining. When serum was isolated, blood was directly harvested in serum separation tubes (Microtainer, BD). Serum was aliquoted and stored at -80°C and thawed prior to use in the ELISA.

Draining lymph nodes (drLN) and spleens were harvested and processed to obtain single-cell suspensions using the plunger of a syringe and a petri dish. Spleens were incubated with ACK lysing buffer (Lonza) for 3 min at room temperature to lyse the red blood cells. Cells were rinsed with PBS containing 1% FBS and 4 mM EDTA prior to staining or washed with complete RPMI prior to *in vitro* cell culture.

### T cell activation assay

PBMC or cells from LN and spleen were plated at 1.5 – 2 x 10^5^ cells per well in 96-well u-bottom plates. As positive control, cells were stimulated with 1 µg/ml anti CD3 (clone 145-2C11) and anti-CD28 (clone 37.51), both Biolegend, Inc. 2 µg/ml of spike protein (LakePharma, Inc) or a peptide mix (1 µg/ml) spanning the S1 and S2 regions of the spike protein, were used to assess the antigen-specific responses (JPT Peptide Technologies, GmbH). To reveal upregulation of CD40L in response to antigen, a blocking anti-mouse CD40 antibody (clone HM40-3) and an APC-conjugated CD40L antibody (clone MR1) were present in the culture for the duration of the assay (29–31). Cells were stained for surface and intranuclear markers after 16-20 hrs.

### Proliferation assay

Mononuclear cells isolated from blood, LN or spleen, were incubated in absence of or with spike protein or spike peptide pools in 96 well plates. After 3 days, 5-ethynyl-2’-deoxyuridine (EdU) was added at 2 µM to the culture medium. Cells were labeled for 16 hrs. Plates were spun down and supernatants were collected for analysis by ELISA and cells were pelleted for staining. Cells were washed twice with PBS prior to labeling with a viability dye. Cells were then washed with 1% BSA in PBS and surface labeled. The pellet was then fixed with 4% PFA for 20 min in 100 µl. After an additional wash with 1% BSA/PBS, cells were washed with a saponin-containing wash buffer and incubated for 20 min in 100 µl. The cells were pelleted again and resuspended in 50 µl of Click-iT reaction cocktail (PBS with Cu_2_SO_4_, Alexa647-Azide and the reducing agent sodium ascorbate). After 30 min of incubation at RT in the dark, cells were washed with wash buffer and intracellular antibodies were added in 30 µl/well. Cells were washed in wash buffer and resuspended in 250 µl PBS/1%BSA prior to analysis on a flow cytometer.

### Antibodies and flow cytometry

For flow cytometric analysis, cells were washed in PBS, then incubated on ice for 20 with a viability dye (zombie yellow; ThermoFisherScientific) to exclude dead cells. Cells were washed with FACS buffer containing PBS, 1% FCS and 0.01% NaN_3_. Surface antibodies used in the study were: TCRβ (clone H57-597), CD4 (clone RM4-5), CD8 (clone 53-6.7), CD44 (clone IM7), CD62L (clone MEL-14), ICOS (clone C398.4A), PD1 (clone J43), CD40L (clone MR1) CD25 (clone PC61.5), OX40 (clone OX-86), CD19 (clone eBio1D3), CXCR5 (clone 2G8)4. Intracellular proteins were detected with the following antibodies: Foxp3 (clone FJK-16s), Ki67 (clone SolA15), granzyme B (clone NGZB). For tetramer analysis, cells were stained separately with the PE-conjugated tetramer (VNFNFNGL, H-2Kb, NIH tetramer core) for 30 min at RT, followed by surface staining or intranuclear staining, as indicated above. All samples were analyzed on an AttuneNxt flow cytometer (ThermoFisherScientific), and data were analyzed with FlowJo software v10.8.1 (Tree Star).

### Spike and RBD direct ELISA

Nunc Maxisorp 96 well u-bottom plates (Thermo Scientific™, high-binding) were coated with Sars-CoV-2 spike or RBD protein (1-2 µg/ml in 50 µl of PBS) and incubated at 4°C overnight. Plates were washed 6x using an automated plate washer with 0.05% Tween20 in PBS. Plates were blocked with 10% non-fat dry milk blocking buffer (BioRad, in PBS/Tween 0.05%) at 100 µl per well for 2 hours at 37°C. Plates were washed 6x times with PBS/Tween 0.05%. Serum was serially diluted 7-11 times in 50 µl of blocking buffer, across the plate. Binding was performed for 90 minutes on a plate shaker at 300rpm protected from light at room temperature. After 6 washes, a secondary HRP conjugated F(ab’)_2_ fragment goat anti-mouse IgG (H+L) antibody (Jackson ImmunoResearch Laboratories, Inc) was prepared at a 1/6000 dilution in blocking buffer. For IgG1-specific antibody detection, an HRP-conjugated goat anti-mouse IgG, Fcγ subclass 1-specific antibody was used (1:6000, Jackson ImmunoResearch Laboratories, Inc); for IgG2a-specific antibody detection, an HRP-conjugated goat anti-mouse IgG2a antibody was used (1:1000, ThermoFisherScientific). The antibody was incubated for 30 minutes on a plate shaker at 300 rpm at room temperate. Plates were washed 6x times and Sureblue™ TMB substrate (VWR) was added at 50 µl/well and allowed to develop for 3 to 5 minutes. The reaction was stopped with 25 µl of H_2_SO_4_. Plates were read at 450 nm absorbance. Three- or four-fold serial dilutions were analyzed and graphed in Prism. EC50 values were calculated using a non-linear regression analysis.

### Cytokine ELISA

Nunc Maxisorp 96 well flat-bottom plates (Thermo Scientific, high-binding) were coated with the monoclonal antibody AN18 (IFN-γ) 1A12 (IL-2) or 11B11 (IL-4) all from Mabtech, at 1 µg/ml in PBS at 100 µl per well. The plates were incubated overnight at 4°C. Plates were washed twice with PBS. Plates were blocked for 1 hour with incubation buffer (PBS, 0.05% Tween 20, and 0.1% BSA) at room temperature. Plates were washed 6x on an automated plate washer with PBS/0.05%Tween 20. The recombinant mouse IFN-γ standard was serially diluted, in incubation buffer, starting at 5 µg/ml, and incubated in 100 µl/well for 2 hours at RT (IL-2 and IL-4 were added at 4 and 1 µg/ml, respectively). The secondary biotinylated antibody R4-6A2 (IFN-γ) 5H4 (IL-2) or BVD6-24G2 (IL-4) were added at 0.5 µg/mL in incubation buffer, after washing the plates, then incubated for 1 hour at RT. After 6 washes, Streptavidin-HRP (BD) was added at 1:250 in incubation buffer and incubated on the plate for 1 hour. For development, Sureblue™ TMB substrate (VWR) was added at 100 µl/well and developed for 5-10 minutes. The reaction was stopped with 25 µl of 0.2M H_2_SO_4_. Plates were read at 450 nm absorbance. Cytokine concentrations in the cell cultures were extrapolated from the standard curve and graphed in Prism.

### T cell isolation and CFSE assay

Total T cells were freshly isolated from pooled splenocytes (pooled by treatment group) using the Easysep negative mouse T cell isolation kit (#19851, Stemcell Technologies). Isolation was performed according to the manufacturer’s protocols. (Stemcell Technologies). After T cell isolation, cells were washed in PBS and labeled with 0.5 µM CFSE diluted in PBS. T cells were cocultured with splenocytes, isolated from C57BL/6 animals and irradiated at 5000 rad prior to pulsing with whole spike protein or S1 and S2 peptide pools. Anti-CD3 and anti-CD28 served as positive control. Cells were cultured in complete RPMI for 3 days, then harvested and stained, prior to analysis on a flow cytometer. CFSE low cells indicate the proportion of proliferating cells.

### Statistical analysis

Statistical analysis was performed with GraphPad Prism v9 software (GraphPad). The *p* values were calculated with a Student paired *t* test (for comparison between two groups), or a one way ANOVA for multiple comparisons. A *p* value <0.05 was considered significant. Error bars denote ± SEM as indicated. The number of biological replicates (individual animals) for each experiment is indicated in the figure legends. The titer for each mouse was calculated as log_10_EC50.

## Data availability statement

The original contributions presented in the study are included in the article/**Supplementary Material**. Further inquiries can be directed to the corresponding author.

## Ethics statement

The animal study was reviewed and approved by the Institutional Animal Care and Use Committee, Earle A. Chiles Research Institute, Providence Cancer Institute.

## Author contributions

AW, RD and H-MH initiated and AW and RD supervised the study. RD and AW designed the experiments. RD and MB performed the experiments. RD, MB and AW analyzed the data. SAbb, SAbr, NJ, AT and AG designed, generated and provided the saRNA-LNP. SJ and BF provided reagents and protocols for the antibody assays. RD and AW wrote the manuscript. All authors contributed to the article and approved the submitted version.

## Funding

This work was supported by the Providence Portland Medical Foundation.

## Acknowledgments

We thank the Earle A. Chiles Research Institute vivarium staff for assistance with injections and husbandry of animals on these studies. We thank Newsha Arezi and Helena Son for their support with formulation of lipid nanoparticles and Sitalakshmi Thampatty and Emily Soon for their support in design and synthesis of saRNAs. We also thank the National Institutes of Health Tetramer Facility for generating the H-2K(b) tetramer for use within the study presented in this manuscript. Marie-Eve Koziol at SEPPIC, Inc. provided the Montanide ISA 51.

## Conflict of interest

AW is founder of AgonOx, which has an ownership interest in OX40 patents. Authors SAbb, SAbr, NJ, AT and AG are/were employed by Precision Nanosystems (PNI).

The remaining authors declare that the research was conducted in the absence of any commercial or financial relationships that could be construed as a potential conflict of interest.

## Publisher’s note

All claims expressed in this article are solely those of the authors and do not necessarily represent those of their affiliated organizations, or those of the publisher, the editors and the reviewers. Any product that may be evaluated in this article, or claim that may be made by its manufacturer, is not guaranteed or endorsed by the publisher.
