# Supplementary material for: OX40 agonist stimulation increases and sustains humoral and cell-mediated responses to SARS-CoV-2 protein and saRNA vaccines
Source: Front Immunol. 2022 Sep 27;13:896310. doi: 10.3389/fimmu.2022.896310 (PMC9551348; doi:10.3389/fimmu.2022.896310)
Supplement: Supplementary file 1 [file DataSheet_1.docx]

Supplementary Material

# Supplementary Figures


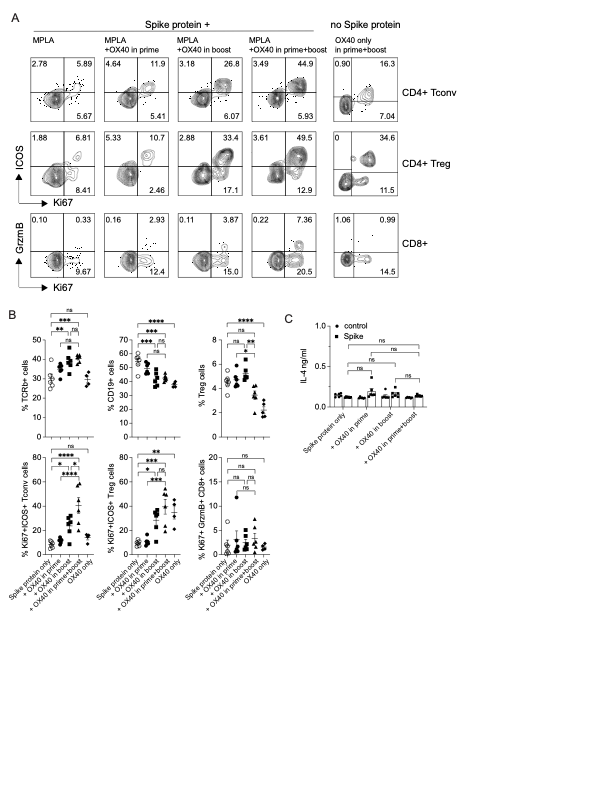


**Supplementary Figure 1.** T cell activation 5 days post boost immunization

Activation of CD4 Tconv, Treg and CD8 T cells was analyzed after the boost, 5 days after the immunization on day 28 (=day33) in groups that received spike protein with MPLA in the prime only, OX40L:Ig in the prime or boost and OX40L:Ig in prime and boost; OX40L:Ig alone served as a control. Experimental scheme and groups are outlined in **Figure 1A,B**. **(A)** Flow cytometric assessment of the activation of Tconv and Treg cells by Ki67 and ICOS or Ki67 and GrmzB expression on CD8 T cells. Numbers in each quadrant indicate percent positive cells. **(B)** Summary of the frequencies of TCRβ+, CD19+ and Treg cell subsets in all groups (top). Summary of the frequencies of Ki67+ICOS+ Tconv, Treg and Ki67+GrzmB+ CD8 T cells in all groups (bottom). **(C)** Supernatants of the 3-day cultures (blood) were assessed for the presence of IL-4 in absence or presence of spike protein. N=6 animals per group, 1 of 2 experiments is shown. Each individual symbol in the bar graphs represents a single mouse. Bar graphs show the mean frequencies ± SEM per group. One-way ANOVA with Tukey’s multiple-comparisons test. *P<0.05, **P<0.01, ***P<0.001, ****P<0.0001, ns = not significant.


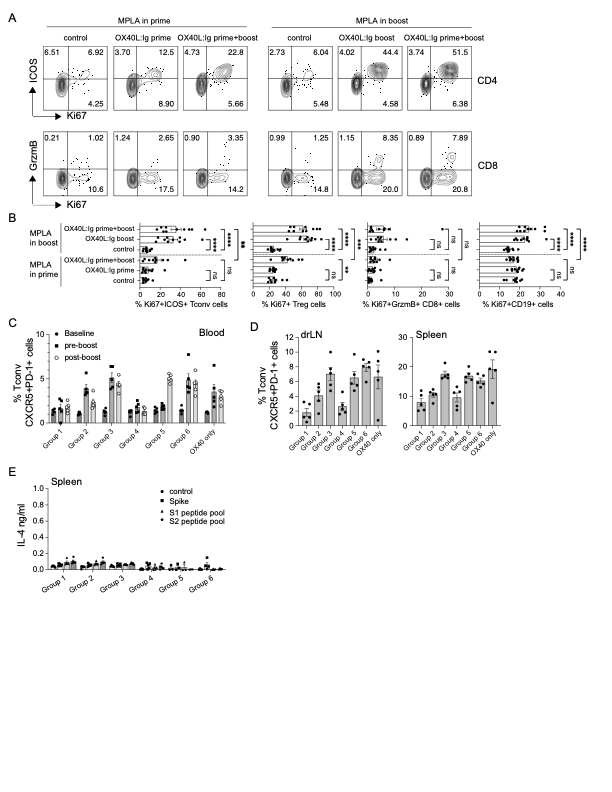


**Supplementary Figure 2.** T cell activation in mice immunized with MPLA in prime or boost

Experimental scheme and groups are outlined in **Figure 2A,B**. **(A)** Flow cytometric assessment of the activation of CD4 T cells by Ki67 and ICOS or Ki67 and GrmzB expression on CD8 T cells at day33, 5 days after boost immunization. Numbers in each quadrant indicate percent positive cells. **(B)** Summary of Tconv, Treg, CD8 T cells and B cells for markers of activation from 2 independent experiments (N=10 animals per group). One-way ANOVA with Tukey’s multiple-comparisons test. *P<0.05, **P<0.01, ***P<0.001, ****P<0.0001, ns = not significant. **(C)** Percentage of CXCR5+ PD-1+ circulating Tfh cells present in blood in each group at baseline, pre-boost and 10 days post-boost. **(D)** Percentage of Tfh cells present in drLN and spleen several weeks after the boost. **(E)** IL-4 in the supernatant of splenocytes cultured for 3 days alone, in presence of spike protein, S1 or S2 peptide pools. Each individual symbol in the bar graphs represents a single mouse. Bars indicate mean ± SEM. 1 of 2 experiments is shown.

**Supplementary Figure 3.** Antibody production in mice immunized with spike protein and MPLA in the prime only, OX40L:Ig in the prime, OX40L:Ig in the boost or OX40L:Ig in both prime and boost. Experimental scheme and groups are outlined in **Figure 1A,B**. Mice were bled on day 28, prior to the boost (indigo circles); day 49, 2 weeks post-boost (red squares); day136, 20 weeks post-boost (blue triangles), and antibody titers to the spike protein were assessed. D=day **(A)** shows total IgG titers against the spike protein, **(B)** shows total IgG titers against the RBD protein. Values on the y-axis indicate absorbance measured at 450 nm. **(C)** Summary of the log_10_(EC_50_) values of all groups at each timepoint. One-way ANOVA with Tukey’s multiple-comparisons test. *P<0.05, **P<0.01, ***P<0.001, ****P<0.0001, ns = not significant

**
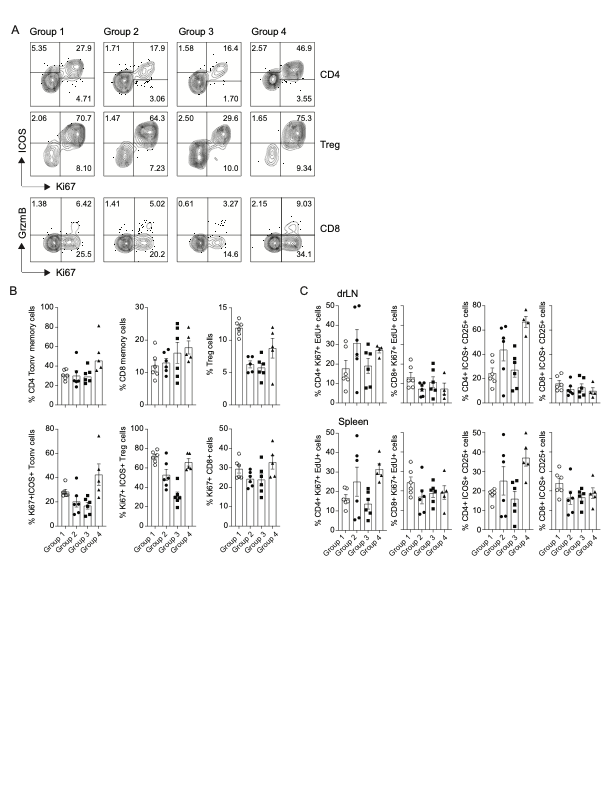
**

**Supplementary Figure 4.** T cell activation 5 days post re-boost immunization

Activation of CD4 Tconv, Treg and CD8 T cells was analyzed 5 days post the booster injections, at day 220. Experimental scheme and groups are outlined in **Figure 4A,B**. **(A)** Flow cytometric assessment of the activation of Tconv and Treg cells by Ki67 and ICOS or Ki67 and GrmzB expression on CD8 T cells. Numbers in each quadrant indicate percent positive cells. **(B)** Summary of the frequencies of memory CD4 and CD8 T cells as well as Treg cells after the re-boost. **(C)** Summary of the numbers of proliferating (EdU+ Ki67+) and activated (CD25+ ICOS+) cells among drLN and spleen after a 3-day culture in presence of spike protein. N=5-6 animals per group. Bar graphs show the mean frequencies ± SEM per group.

**
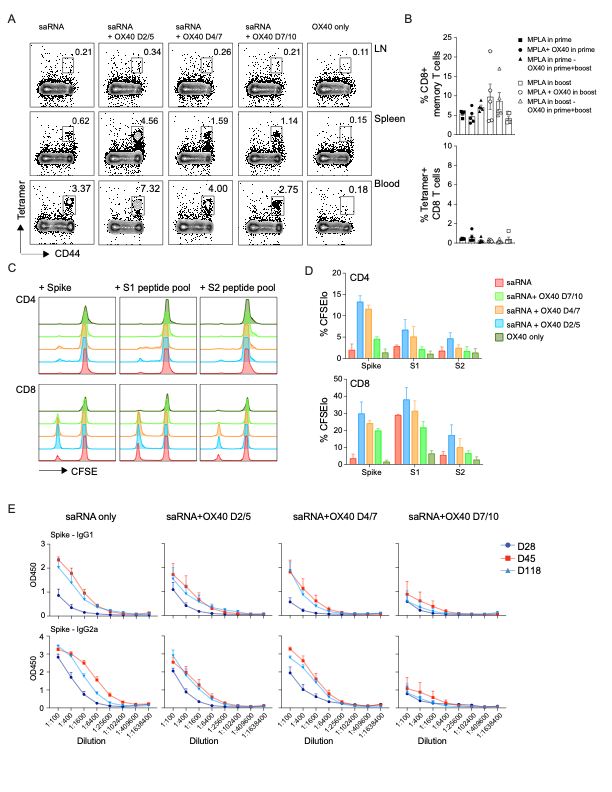
**

**Supplementary Figure 5.** Spike-specific, cytotoxic CD8 T cells are increased after saRNA-LNP vaccination

Experimental scheme and groups are outlined in **Figure 5A,B.** D=day **(A)** Flow cytometric assessment of the frequency of tetramer+ CD44+ CD8 T cells 12 weeks post boost (day118). Each plot represents one animal per group in LN, spleen and blood. The outlined box indicates the tetramer+ cells. **(B)** Frequencies of memory (CD62L^lo^CD44^hi^) CD8 T cells and tetramer+ CD8 T cells were assessed in animals which received the spike-protein vaccine as indicated in **Figure 2A,B**. Animals were bled at day107. Histograms show the mean frequencies ± SEM per group. Each data point represents a separate animal. **(C)** CFSE-labeled T-cells, isolated from splenocytes from all groups on day124, were cultured with irradiated APCs alone, pulsed with spike protein, or S1 and S2 peptide pools. Cell divisions were analyzed by flow cytometry after 4 days in CD4 and CD8 T cells. **(D)** Histograms show the percentage of CFSE^lo^ cells in CD4 and CD8 T cells in 4-day cultures with antigen. N=5 in A and N=10 in C-D. Colors match groups. 1 of 2 experiments is shown. **(E)** Antibody titers of groups which received saRNA in LNP only, or saRNA-LNP with OX40L:Ig at days 2/5, days 4/7 or 7/10. Mice were bled on day 28, prior to the boost (indigo circles); day 45, 2 weeks post-boost (red squares); day118, 16 weeks post-boost (blue triangles), and antibody titers to the spike protein were assessed. The graphs show IgG1 titers (top row) and IgG2a titers (bottom row) against the complete spike protein. Values on the y-axis indicate absorbance measured at 450 nm.
